# *Plasmodium vivax* in vitro continuous culture: the spoke in the wheel

**DOI:** 10.1186/s12936-018-2456-5

**Published:** 2018-08-20

**Authors:** Maritza Bermúdez, Darwin Andrés Moreno-Pérez, Gabriela Arévalo-Pinzón, Hernando Curtidor, Manuel Alfonso Patarroyo

**Affiliations:** 1 Receptor-ligand Department, Fundación Instituto de Inmunología de Colombia (FIDIC), Carrera 50 No. 26-20, Bogotá, Colombia; 2 Molecular Biology and Immunology Department, Fundación Instituto de Inmunología de Colombia (FIDIC), Carrera 50 No. 26-20, Bogotá, Colombia; 3 Livestock Sciences Faculty, Universidad de Ciencias Aplicadas y Ambientales (U.D.C.A), Calle 222 No. 55-37, Bogotá, DC Colombia; 4 Basic Sciences Department, School of Medicine and Health Sciences, Universidad del Rosario, Carrera 24 No. 63C-69, Bogotá, DC Colombia

**Keywords:** *Plasmodium vivax*, Reticulocyte, In vitro culture, Receptor, Ligand

## Abstract

Understanding the life cycle of *Plasmodium vivax* is fundamental for developing strategies aimed at controlling and eliminating this parasitic species. Although advances in omic sciences and high-throughput techniques in recent years have enabled the identification and characterization of proteins which might be participating in *P. vivax* invasion of target cells, exclusive parasite tropism for invading reticulocytes has become the main obstacle in maintaining a continuous culture for this species. Such advance that would help in defining each parasite protein’s function in the complex process of *P. vivax* invasion, in addition to evaluating new therapeutic agents, is still a dream. Advances related to maintenance, culture medium supplements and the use of different sources of reticulocytes and parasites (strains and isolates) have been made regarding the development of an in vitro culture for *P. vivax*; however, only some cultures having few replication cycles have been obtained to date, meaning that this parasite’s maintenance goes beyond the technical components involved. Although it is still not yet clear which molecular mechanisms *P. vivax* prefers for invading young CD71^+^ reticulocytes [early maturation stages (I–II–III)], changes related to membrane proteins remodelling of such cells could form part of the explanation. The most relevant aspects regarding *P. vivax* in vitro culture and host cell characteristics have been analysed in this review to explain possible reasons why the species’ continuous in vitro culture is so difficult to standardize. Some alternatives for *P. vivax* in vitro culture have also been described.

## Background

Continuous in vitro *Plasmodium falciparum* culture (standardized in the 1970s) [1–4] has been an indispensable tool for understanding the parasite’s life cycle and identifying most proteins involved in erythrocyte invasion, some of which have been tested as vaccine components at clinical level [1–6]. Developing a methodology enabling the continuous growth and propagation of *Plasmodium vivax* (*P. vivax* being the second most important species causing malaria in humans) has thus become a challenge for several research groups studying this parasite [7–13].

Unfortunately, maintaining a continuous culture of *P. vivax* in vitro is still difficult, despite different aspects having been studied and modified, i.e. different culture media [13, 14], parasite [9, 15] and reticulocyte [7, 10] sources, added to the different methods for obtaining and enriching invasion target cells [16]. It has only been possible to maintain a culture in vitro for up to 26 months to date, having < 0.1% parasitaemia [14], which might be due to merozoites (Mrz) losing their ability to re-invade new host cells [11, 13, 14, 17, 18].

The forgoing has discouraged research orientated towards knowing in detail the mechanism used by *P. vivax* for specifically invading reticulocytes; consequently, there has been a delay in identifying new molecules, the function they fulfil and their antigenic and immunogenic capability; such information is essential for selecting specific proteins to be included when developing parasite control methods.

This work has been aimed at reviewing aspects which have been taken into account for standardizing an in vitro *P. vivax* culture and proposes some alternatives which could be considered.

### The current state of *Plasmodium vivax* biology

*Plasmodium vivax* is a parasite causing malaria in humans; it has been included on the international health agenda regarding its early eradication, mainly due to the high morbidity rates it causes and its wide geographical distribution [6]. This parasite species displays particular biological characteristics, such as hypnozoite development in the liver and rapid gametocyte formation. Interestingly, the parasite exclusively infects immature erythrocytes (reticulocytes), representing just 1–2% of total red blood cells (RBC) from adult human peripheral blood. These cells are fragile, have rapid maturation and complex procedures are required for obtaining enriched samples, hence maintaining a *P. vivax* continuous culture in vitro is extremely difficult [16].

**Fig. 1:**
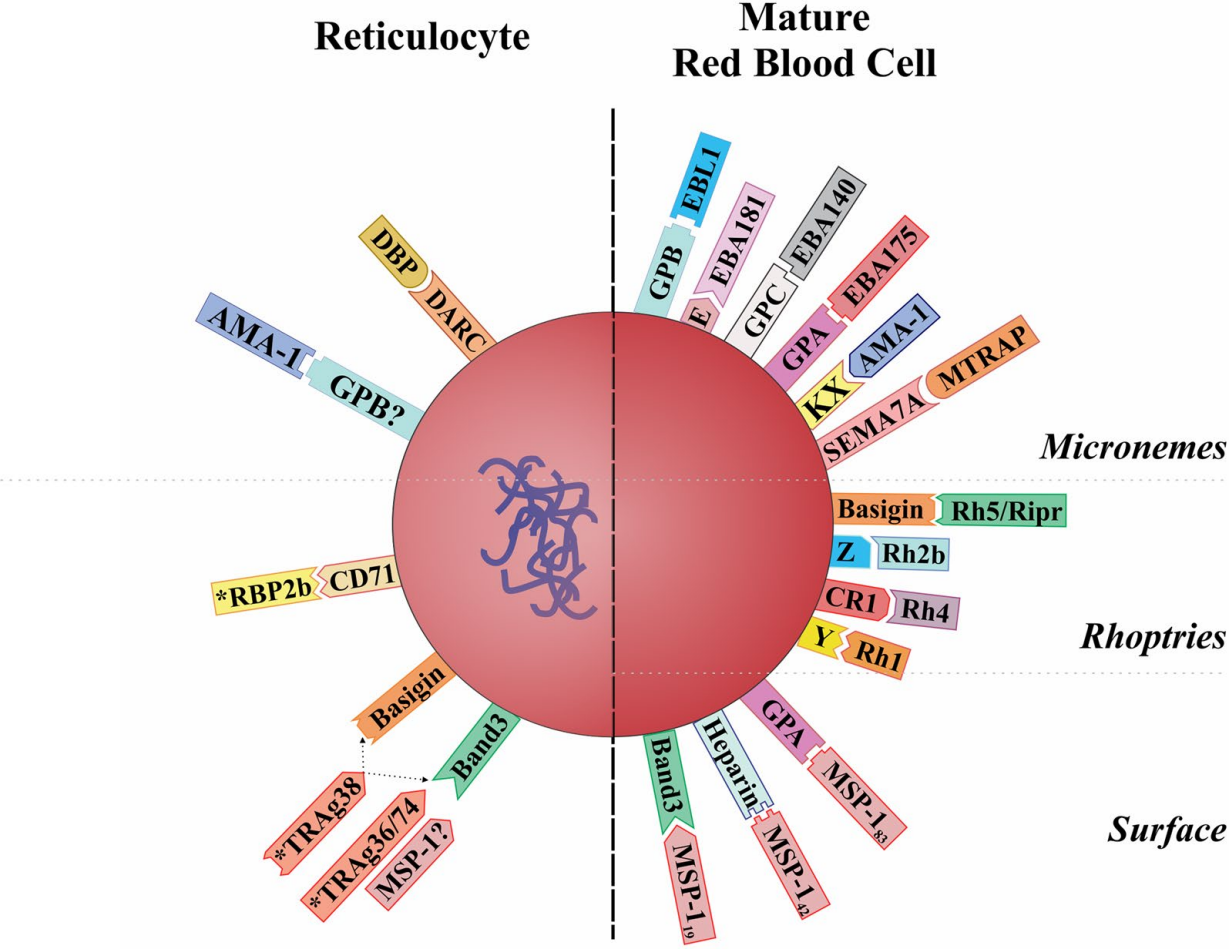
*Plasmodium vivax* and *P. falciparum* merozoite host cell adhesion proteins. The figure shows the *P. vivax* (left-hand side) and *P. falciparum* (right-hand side) proteins described to date having a binding-related function regarding receptors identified on target cells: reticulocyte and mature erythrocytes, respectively. Mrz proteins’ subcellular localization is indicated. An asterisk indicates those molecules with unknown subcellular localization

The absence of an in vitro culture in *P. vivax* could be considered as “the spoke in the wheel” which has caused a considerable delay (between 5 and 10 years) in executing certain types of studies, such as omic sciences, invasion inhibition and determining adhesin-type ligands, epitopes and antigens [19–21], i.e. compared to those for *P. falciparum* [22–24]. In fact, more than 50 proteins involved in *P. falciparum* binding to and invading target cells have now been described as well as some receptors for them [25–28]. By contrast, only 23 proteins associated with *P. vivax* invasion of reticulocytes have been characterized (using parasites from patients [29–31] and infected animals’ samples [32–35]) and few receptors have been studied (Fig. 1). The proteins characterized to date have been tryptophan rich antigens (*Pv*TRAg26.3, *Pv*TRAg33.5, *Pv*TRAg34, *Pv*TRAg35.2, *Pv*TRAg36 (band 3 as receptor) [36], *Pv*TRAg36.6, *Pv*TRAg38 (basigin [37] and band 3 [38] as receptors), *Pv*TRAg40, *Pv*TRAg69.4, *Pv*TRAg74 (band 3 as receptor) [36], rhoptry neck protein 5 (RON5) [39], reticulocyte-binding proteins RBP-1a, RBP-1b [40], RBP-2b (CD71 as receptor) [41, 42], erythrocyte binding protein 2 (EBP-2) [43], GPI-anchored micronemal antigen (GAMA) [44], reticulocyte binding surface antigen (RBSA) [45], the Duffy binding protein (DBP) (DARC as receptor) [46, 47], reticulocyte binding protein 1 (RBP-1) [48], merozoite surface protein 1 (MSP-1) (possible receptor, band 3) [49], apical membrane antigen 1 (AMA-1) (chymotrypsin- and neuraminidase-sensitive receptor, GPB?) [50] and rhoptry neck proteins 2 and 4 (RON2 and RON4) [51].

The small list of characterized ligands and receptors reveals the tremendous challenge faced by researchers considering studying *P. vivax* in terms of continuous propagation to understand different aspects of the parasite’s basic biology. In view of this and aimed at making significant advances in clinical and basic research regarding the species, several groups have focused on standardizing a continuous in vitro culture system for *P. vivax* blood stages for which some essential parameters for optimizing parasite growth and development have been determined.

### Culturing *Plasmodium vivax*

**Fig. 2:**
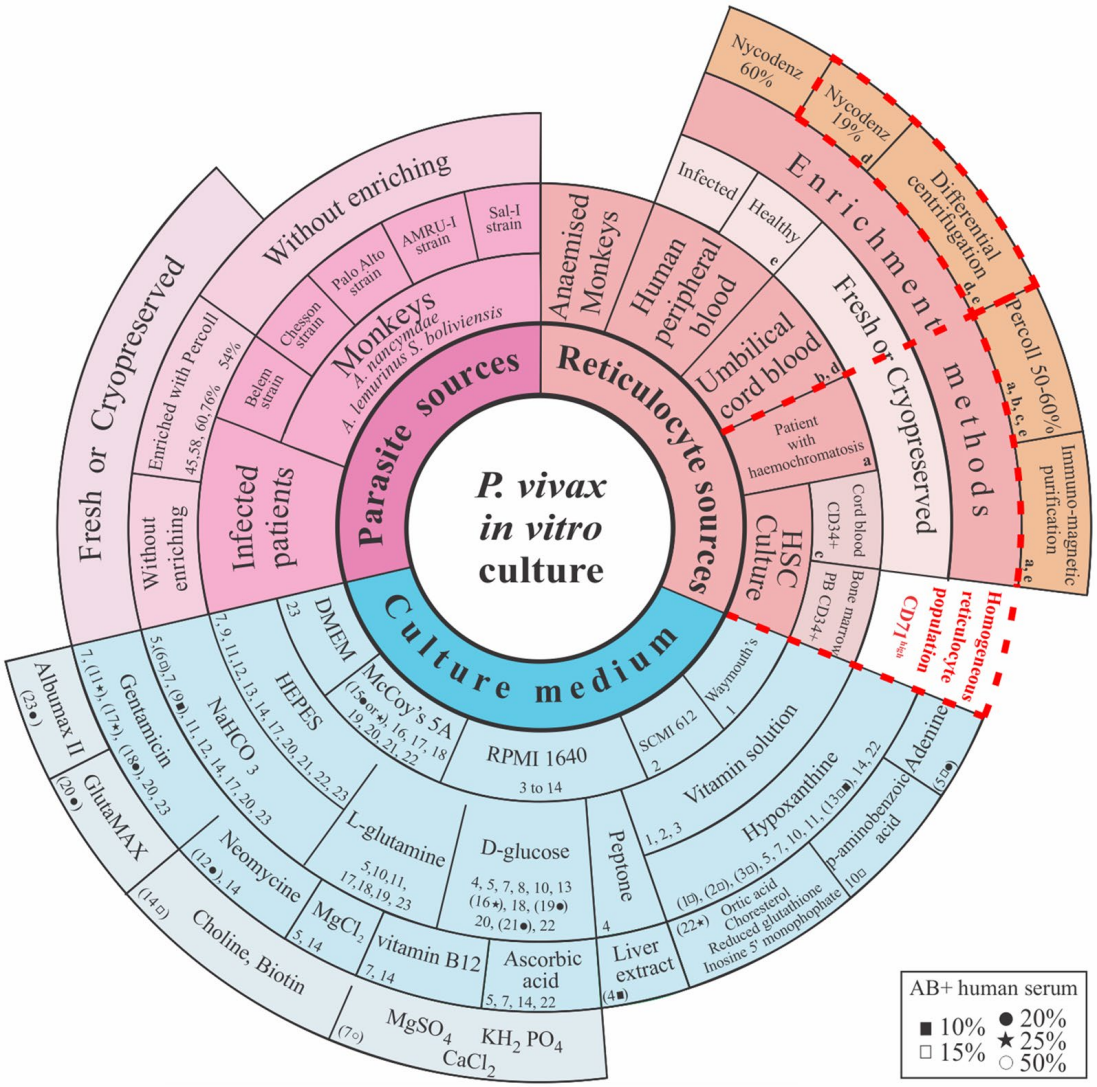
Conditions evaluated for culturing *Plasmodium vivax* in vitro. The figure shows modifications made to culture medium, parasite sources and the target cells which have been used in culturing the *P. vivax* parasite. Each combination evaluated is described in the culture medium section. For example, 14 indicates RPMI 1640 medium, which contains HEPES, NaHCO_3_, neomycin, vitamin B12, hypoxanthine, ascorbic acid, MgCl_2_, biotin, choline and 15% AB^+^ human serum (box showing typology). The reticulocyte source section lists these cells’ enrichment methodology using the letters a–e. Sections of the graphic enclosed by a red discontinuous line indicate the best target cell sources and/or enrichment methods available so far

The first reports about culturing malarial Plasmodium date from the beginning of the twentieth century, some techniques being more controversial than others (i.e. parasite culture from infected water and keeping parasites alive in milk for several days) [52]. Although the first successful *P. vivax* in vitro culture was reported in 1912 [53, 54], a base protocol for propagating this parasite species was only established at the end of the 1970s [4, 55, 56]. Since then, one or more of the factors involved in the culture have been modified in various attempts at finding an efficient methodology (Fig. 2). However, it has not been possible to date to maintain a culture, given two main problems: parasitaemia dynamics and the amount of days for maintaining a *P. vivax* in vitro culture. Although is not clear why *P. vivax* Mrz in culture lose their ability to re-invade new host cells, the isolate or parasite strain and target cells may have intrinsic characteristics which can influence *P. vivax* propagation (despite modifications to the culture media). The factors related to maintaining a *P. vivax* in vitro culture (i.e. culture media, parasite and reticulocyte origin) will therefore be analysed.

### Culture media and supplements

Several media and supplement combinations have been tested to ensure the conditions and nutrients required for optimum *P. vivax* growth (Fig. 2). The first reported medias for *P. vivax* in vitro culture were modified Harvard, RPMI-1640, Waymouth’s and SCMI 612 supplemented media [4, 53, 57] (Fig. 2); it was seen that only SCMI 612 medium improved parasite viability [58] (Table 1). Other research showed that RPMI 1640 medium supplemented with MgCl_2_ [59], ascorbic acid, hypoxanthine, vitamin B12, choline and biotin [60] improved parasite maturation. However, in later studies in which RPMI 1640 medium was used [12, 56, 61–65], even in mixture with different compounds and salts (MgS0_4_, KH_2_P0_4_ and CaCl_2_) and 50% AB^+^ human serum, there was no improvement in parasitic density, suggesting that the RPMI 1640 media is not appropriate for *P. vivax* continuous growth and development [65].

**Table 1:** Studies related to *Plasmodium vivax* in vitro culture development

Date	Parasite source	Reticulocyte source	Type of culture	Culture period [days or cycles (when explicitly stated)]	Contribution	Refs
1912	Infected patients	Human erythrocytes	Short-term	8	The first attempt to culture *P. vivax*. Defibrinated or citrated human blood seemed to be the most favourable culture medium	[53]
1913	Infected patients	Human erythrocytes	Short-term	8	This showed that *P. vivax* could be grown at 39 °C	[54]
1947	Infected patients	Human erythrocytes	Short-term	8	Tissue cultures were made from the fatty layer and from the buffy coat after it had been clotted with chick embryo extract	[86]
1979	Vietnam Palo Alto strain from *Aotus* monkeys and infected patients	Monkey red blood cell fraction	Short-term	8	First culture of *P. vivax* derived from on-going infection in *Aotus monkeys*	[57]
1981	Infected patient	Human RBC	Long-term	43	The first report of maintaining *P. vivax* for 20 cycles	[8]
1984	Infected patients	Human erythrocytes	Short-term	4	Magnesium chloride was important as culture supplement	[59]
1985	Infected patients (43 isolates)	Human RBC	Short-term	6	Three different culture media were used: better results were obtained with SCMI 612 than RPMI 1640 and/or Waymouth	[58]
1987	Belem strain from *Saimiri* monkeys enriched with Percoll (54%)	Reticulocyte-enriched human peripheral blood with Percoll (65%)	Invasion assay	Not provided	*P. vivax* Mrz invasion depends on Fy6 presence	[70]
1988	Infected patients. 8 isolates enriched with Nycodenz (58–60%)	UCB, bone marrow and human peripheral blood from haemolytic anaemia patients	Short-term	4	The parasite was concentrated during ring and trophozoite stages to improve invasion rate	[60]
1988	Palo Alto strain from *Aotus nancymaae*	Monkey blood after artificially induced anaemia	Invasion assay	5 cycles	Parasites were grown in continuous shaking conditions to increase Mrz contact with target cells	[61]
1989	Belem strain from monkeys enriched with Percoll (54%)	Reticulocyte-enriched human peripheral blood and monkey blood with Percoll (65%)	Invasion assay	Not provided	Confirmed the role of the Duffy blood group antigen as a ligand for *P. vivax* Mrz	[62]
1991	Infected patients	Human erythrocytes	Short-term	15	Good growth in the presence of liver extract	[63]
1991	Infected patients	Human erythrocytes	Short-term	4	Parasite density doubled after 96 h	[64]
1992	Chesson strain in *Saimiri* monkeys	Reticulocyte-enriched human and monkey blood by Percoll/Renografin-60	Short-term	22	The flow-vessel system was the best method available at the time	[56]
1997	Chesson strain in *Aotus nancymaae* and *A. lemurinus griseimembra*	Reticulocyte-enriched haemochromatosis blood by homologous plasma differential centrifugation or Percoll (60%)	Short-term	15	This study described the differential centrifugation as an effective method for host cell enrichment. Blood from haemochromatosis patients may be invaded easily by *P. vivax*	[7]
2000	Infected patients	Human cord	Long-term	52	Parasite re-invasion was maintained for 7 to 8 days	[87]
2001	Infected patients suffering acute infection	None	Short-term	12	The *P. vivax* culture was maintained without adding fresh reticulocytes to the medium	[65]
2007	Infected patients (15 isolates)	UCB and patients with haemochromatosis	Long-term	40	Cultures supplemented with haemochromatosis patients’ reticulocytes were maintained for a longer time than those supplemented with UCB	[12]
2007	Infected patients. 7 isolates enriched with Percoll (60%)	Culturing HSC-derived reticulocytes enriched with Percoll (50-60%)	Long-term	85	Parasites could invade nucleated cells and erythroblasts which are mostly found in bone marrow	[10]
2011	Infected patients [schizonts enriched with Percoll (45%)]	Reticulocytes enriched from UCB with Percoll (70%)	Invasion assay	2 cycles	A new protocol for culturing *P. vivax* in laboratories located in endemic countries was developed	[11]
2012	Acute infection and cryopreserved isolates	Reticulocytes enriched from UCB with Percoll (70%)	Short-term	10	It was shown that cryopreserved samples (parasites and reticulocytes) could be used for invasion and initiate short-term culture	[18]
2012	Infected patients. Isolates cryopreserved for 3 years	Culturing HSC-derived reticulocytes (cryopreserved for 1 year)	Invasion assay	Not provided	HSC-derived reticulocytes could guarantee a more homogenous and standardized reticulocyte population	[9]
2012	Infected patients	The same infected patients	Short-term	3	Wild isolates were preserved in wet ice for 9–10 days	[13]
2013	Infected patients	Culturing HSC-derived CD34^+^ from bone marrow or human peripheral blood	Short-term	Not provided	It was shown that CD34 + hHSC from peripheral blood and bone marrow could be expanded and differentiated to reticulocytes using a novel stromal cell. It was suggested that the absence of fetal haemoglobin could improve *P. vivax* invasion	[66]
2014	AMRU-I strain in *Aotus nancymaae*	Culturing HSC-derived CD34^+^ from UCB (these were cryopreserved after 8 days culture)	Short-term	4	A substantial amount (up to 0.8% of the cells) of newly invaded reticulocytes was obtained 24 h after initial culture	[17]
2015	Infected patients (30 isolates)	HSC culture, reticulocyte enriched peripheral blood [with Nycodenz (19%)] and SCU	Long-term	780	The only study to date which has managed to maintain the culture for a prolonged time (26 months), with 0.01%. parasitaemia. Reticulocytes obtained from adults’ peripheral blood and enriched on Nycodenz seemed to improve parasite maturation conditions, as well as gametocytes obtained in four of the cultured isolates. Nycodenz had no notable toxic effects on cells and was thus appropriate for enriching them and favoured parasite invasion during long-term *P. vivax* infection	[14]
2015	Infected patients (15 isolates)	UCB	Short-term	Not provided	McCoy’s 5A medium supplemented with l-glutamine, HEPES buffer, NaHCO_3_, hypoxanthine, 0.5% Albumax II (a new compound) and gentamicin was useful for culturing *P. vivax*	[67]
2016	Sal-1strain in *Aotus lemurinus*	Haemochromatosis patients or buffy packs enriched with modified differential centrifugation, Percoll (70%) or CD71^+^ coupled immunomagnetic bead-based purification method	Short-term	14	Reticulocytes enriched by differential centrifugation in homologous plasma (20%) were more apt to be invaded by *P. vivax* parasites. GlutaMAX did indeed improve parasite viability, growth and development compared to traditional l-glutamine	[15]
2017	Infected patients (cryopreserved isolates)	*Saimiri boliviensis* and human blood	Long-term	233	Parasites could re-invade monkey and human erythrocytes. Dulbecco’s Modified Eagle Medium (DMEM) was effective for *P. vivax* culture	[69]

McCoy’s5A medium has also been routinely used [7, 9–11, 13, 14, 17, 18, 66, 67] in combination with various supplements such as d-glucose and l-glutamine, or just with 20% or 25% AB^+^ human serum [9, 10, 17, 18]. It has been reported that a medium consisting of McCoy’s5A supplemented with HEPES, NaHCO_3_, d-glucose, gentamycin and 50% AB^+^ human serum maintains parasite density (10 parasites/µL) during the first 5 days of culture. However, such parasite density can be maintained after 5 days using just media supplemented with 25% AB^+^ human serum [14]. Two compounds improving parasite development in McCoy’s5A medium have been reported recently: Albumax II [67] and GlutaMAX [15, 68] (Fig. 2, Table 1). GlutaMAX (l-alanyl-l-glutamine dipeptide) did indeed improve parasite viability, growth and development compared to l-glutamine as this compound does not break down to form toxic by-products, such as ammonia, formed by traditional l-glutamine [15]. This highlighted the fact that *P. vivax* could be very sensitive to the accumulation of waste or toxic products in in vitro conditions.

The use of Dulbecco’s Modified Eagle Medium (DMEM) for *P. vivax* culture supplemented with l-glutamine, HEPES and hypoxanthine has been reported recently. Parasitaemia was maintained for 233 days and was ended because of bacterial contamination [69]. The fluctuation in parasitaemia using DMEM was similar to that observed when the parasite has been grown in McCoy’s5A medium [14], suggesting that these media (McCoy’s 5A and DMEM) are useful for culturing and maintaining parasite maturation and replication in vitro. Future trials should be conducted with McCoy’s5A or DMEM medium, supplemented with 25% human serum (with Glutamax and Albumax) to evaluate whether parasite density can be maintained and/or increased in culture.

### Parasite source

The parasite has been used from two sources for standardizing *P. vivax* in vitro culture, i.e. isolated from humans and from primates (Fig. 2, Table 1). Regardless of the source, it has been observed that keeping the culture in static conditions improves culture parasitaemia [15, 61] as well as depleting white blood cell amount in reticulocyte samples, as leukocytes’ phagocytic activity against parasites affects their invasion [53, 61]. Likewise, it has been shown that cryopreservation [9, 18] enables maintaining parasite viability and invasive capability when preserved and stored for days [13, 69] or even years [9].

Difficulty related to variation in both longevity and parasitaemia has occurred regarding in vitro culture with parasites obtained from humans. For example, it has been reported that different isolates could be maintained in culture for several days: i.e. from 10 or 30 days [12], from 2 to 8 days or up to 85 days (more than 2 months) [10]. An in vitro culture of 3 *P. vivax* isolates was recently maintained for more than 1 year (26 months), having ~ 0.01% parasitaemia [14]. Other research has shown that culture parasitaemia can increase almost tenfold when using parasites from isolates which were enriched during ring stage by Percoll gradient [11, 68]. Despite this, the parasite progressively loses its invasion ability, a problem which has not yet been resolved to date. These studies suggested that each *P. vivax* isolate has its own characteristics related to adaptation to in vitro culture and thus their invasion capability, multiplication rate and parasitaemia are variables which must be considered when standardizing a culture for each of them. This hypothesis can be supported by a study by Russell et al., who evaluated umbilical cord blood (UCB) reticulocyte invasion inhibition using 85 *P. vivax* clinical isolates. They found that invasion efficiency was constant for each specific isolate but that 85.79% of the total variance depended on isolate type [11]. Heterogeneity concerning human isolates’ invasion efficacy and *P. vivax* parasitaemia density variation thus makes the methodologies used for culturing the parasite not suitable for studying its biology and further complicates the development of a robust and reliable culture method.

Unlike parasites obtained from humans, primate-adapted *P. vivax* strains can be used to start in vitro culture anytime, given their availability. This is why some research groups have worked with several *P. vivax* strains (Fig. 2, Table 1) [56, 57, 61, 62, 70] which were able to adapt to invade erythroid cells in vitro (from humans suffering from haemochromatosis [7, 15]), owl monkey cells [7] and reticulocytes obtained from the maturation of UCB haematopoietic stem cells (HSC) - CD34^+^ [17], cultures reaching > 0.5% parasitaemia. These results support the notion that monkey-adapted *P. vivax* strains do not lose their capability to invade, regardless of cell source, and therefore, represent a good alternative for establishing a parasite culture.

Considering invasion efficacy variability in cultures from human sources and the great adaptability of strains in monkeys, it can be suggested that the same parasite strain must be used during attempts at standardization to establish the basic and necessary conditions for maintaining a long-term in vitro culture.

### Target cell source

Obtaining reticulocytes for continuous supplementation in culture has been a huge inconvenience since these cells only form 1 to 2% of human peripheral blood, mature quickly, are fragile and have low viability. UCB (containing 6.9–7.9% reticulocytes), peripheral blood from humans or splenectomized monkeys, blood from haemochromatosis patients (14–17% reticulocytes) and HSC (variable reticulocyte percentages) have been used as reticulocyte sources for standardizing an in vitro *P. vivax* parasite culture [7, 12, 61] (Fig. 2, Table 1). Different techniques such as density gradients (Percoll and Nycodenz), ultra-centrifugation and/or immunomagnetic separation have also been used for obtaining a greater percentage of reticulocytes in culture, Nycodenz being one of the most appropriate compounds as it has had no notable toxic effects on cells [7, 14, 15] (Fig. 2).

Although UCB are a good source of reticulocytes, it has been shown that they do not support the parasite’s full development and are easily lysed [61]. Fetal haemoglobin in such erythroid cells apparently produces an inhibitory effect for *P. vivax* growth, equivalent to that reported for *P. falciparum,* which does not grow adequately in erythrocytes containing fetal haemoglobin [71, 72]. The forgoing has been supported since it has been reported that reticulocytes from CD34^+^ erythroid progenitors (derived from adult peripheral blood or bone marrow) and from adults’ peripheral blood lacking fetal haemoglobin could improve *P. vivax* invasion [66] regarding maturation and gametocyte production [14]. Whilst these studies have shown that UCB does not seem to be very suitable for standardizing an in vitro *P. vivax* culture, another study has shown that fetal haemoglobin caused no alteration in parasite growth and up to 0.4% parasitaemia was reached during the first days of culture [11]. Although it is not clear how haemoglobin could alter parasite development, this effect might depend on the reticulocyte’s maturation stages (variability) and the availability of these stages in the UCB source.

Another great concern related to using reticulocytes is their rapid maturation. It has been suggested that these target cells can be frozen to provide a reserve and then used for supplementing a culture when required. Different studies have reported that both fresh reticulocytes and freshly thawed reticulocytes were susceptible to invasion by *P. vivax* Mrz. Interestingly, such susceptibility did not depend on reticulocyte source since they were obtained from UCB [18], haemochromatosis patients (in which the cells were enriched using Percoll gradient [7, 18] or differential centrifugation [7]) and human cord HSCs [9, 17] (enriched by Percoll density gradient [10]). Notably, cryopreserved cells which were then thawed had up to 70% viability and such percentage remained stable compared to that for fresh samples [9].

According to the literature, haemochromatosis patients have been one of the best reticulocyte (fresh or cryopreserved) sources. These reticulocytes, enriched by differential centrifugation in 20% homologous plasma [7, 15], were easily invaded, able to support both parasite growth and invasion [12] and maintain a stable schizont percentage [7]. Although Percoll gradient has been widely used for enriching reticulocytes obtained from haemochromatosis patients (Fig. 2), two studies have reported that cell viability and stability could be affected by damage to or the loss of some membrane receptors which might be essential for *P. vivax* invasion [7, 15]. Despite this, one of the drawbacks of this target cell source is that haemochromatosis mainly occurs in Caucasians, a type of reticulocyte not normally accessible for researchers outside Europe or North America [14]. Using HSC-derived reticulocytes could guarantee a more homogenous and standardized cell population which would enable obtaining a high reticulocyte concentration (> 20%) [9], necessary for maintaining *P. vivax* cultures.

Factors such as culture medium, as well as parasite and reticulocyte sources have been revised and possible modifications which could improve parasite development in vitro have been pointed out. However, target cells must be analysed in depth in relation to their intrinsic characteristics enabling the parasite to invade them.

### Reticulocyte receptors: the new molecular keys?

Taking into account that *P. vivax* Mrz only invade reticulocytes, the next question arises: Which characteristics do reticulocytes have so that *P. vivax* can only invade this type of cell? Immature reticulocytes in bone marrow contain ribonucleic acid (RNA) and undergo different biochemical, biophysical and metabolic changes during their maturation to normocytes within a period of 72 h [73]. The differences between reticulocytes and normocytes have been studied at molecular level in murine and human models [74–76]. It has been found that the main difference between these two cells is the abundance of their receptors, since more than 60% of proteins quantified in immature erythrocytes became reduced (from 2 to 100 times) as they matured to normocytes, whilst around 5% had higher expression levels. Receptors such as transferrin receptor (CD71) on reticulocyte membrane decrease progressively until their total absence in normocytes [77, 78]; this, together with cytoplasmatic RNA (Thiazole Orange stained, TO) concentration, has enabled classifying the reticulocyte population into four groups: Heilmeyer stage I (CD71^high^TO^high^), Heilmeyer stages II and III (CD71^low^TO^med^) and Heilmeyer stage IV (CD7^−^TO^low^) [73, 79].

Most *P. vivax* in vitro culture studies have been restricted to using stage III (the first to emerge from bone marrow), stage IV or mature reticulocyte (CD71^−^TO^low^) populations. Using these two reticulocyte stages and their rapid maturation could provide an explanation for why the parasite loses its infective capability through various replication cycles. This could also explain why less than 1% parasitaemia has only been achieved in most assays performed to date [12, 14]. In line with the forgoing hypothesis, it has been shown that cryopreserved parasite isolates from patients can infect stage I reticulocytes (CD71^high^TO ^high^) representing only 0.02% in total blood [79]. The infected cells mature rapidly and almost completely lose reticular matter 3 h post-invasion, thereby showing that parasite invasion promotes rapid reticulocyte maturation [79, 80]. Similar results have been found by Shaw-Saliba et al., when evaluating a culture of Sal-I strain parasites adapted in *A. lemurinus* monkeys with CD71^high^ reticulocytes. As expected, parasites preferentially invaded stage I reticulocytes (CD71^high^TO^high^) and very few managed to invade stage IV reticulocytes (CD71^−^TO^low^) [15]. Research by Golenda and Udomsangpetch showed that *P. vivax* development and invasion levels were better using haemochromatosis patients’ blood; these results also support the previous hypothesis [7, 12] as people suffering this type of anaemia produce a larger amount of stage I reticulocytes (CD71^high^TO^high^) to balance the decrease of erythrocytes in blood flow [81].

These observations lead to another question: Why can *P. vivax* Mrz only invade the most immature reticulocyte stage? Several studies using different approaches could provide an answer to this question. One such was related to DARC receptor abundance on CD71^high^TO^high^ reticulocytes and conformational changes affecting such receptor enabling the parasite to bind to and invade this type of cell [82]. It has been found that although total DARC protein remains constant throughout reticulocyte maturation there is selective exposure of one DARC amino acid sequence (QLDFEDVWNSSY) by conformational changes before maturation which causes DBP to bind more specifically to CD71^high^/TO^high^ reticulocytes than to other mature reticulocyte or erythrocyte subpopulations [82]. Other studies showing *P. vivax* proteins’ preference for binding to CD71^high^ reticulocytes [44, 45] and evidence about RBP-2b binding to CD71 membrane receptor have been published very recently [42]. The above highlights the most immature reticulocyte stage (CD71^high^ TO^high^) as the molecular key (receptor) which *P. vivax* takes advantage of to invade and replicate within cells. This could suggest that using reticulocytes from bone marrow and/or from patients suffering different types of haemolytic anaemia (i.e. haemochromatosis) could be appropriate for maintaining and developing a continuous in vitro culture system involving *P. vivax* blood stages [83]. However, it would be ethically complicated to work with samples from patients suffering from some type of anaemia, which is why using stage I homogenous reticulocytes (CD71^high^ TO^high^) obtained from HSC could be a viable alternative.

### Others challenges to be faced

It has been demonstrated that *P. vivax* DBP binds more to reticulocytes having the Fya^−^/Fyb^+^ phenotype [84], which could be an advantage regarding parasite culture.

It would be expected that supplementing cultures with a CD71^high^TO^high^ enriched reticulocyte population and having such phenotype should maintain parasitaemia, invasion efficiency and a culture for a long time.

A new challenge today concerns the fact that enough evidence has been amassed to indicate that the parasite can also invade Duffy negative cells (Fya^-^/Fyb^-^) via an as-yet-unknown alternative invasion route [85]. This finding suggests two possibilities; first, such *P. vivax* property remains unknown due to this parasite’s sub-microscopic and asymptomatic parasitaemia and secondly this could be a new adaptation phenomenon where ligand-receptor interaction routes different to DBP-DARC are acting as survival strategy for propagating cells having the Fya^−^/Fyb^−^ phenotype. Studies aimed at ascertaining whether *P. vivax* target cell invasion route is via the RBP2b-CD71 interaction using Duffy negative phenotype CD71^high^ reticulocytes are in need [42]. Future assays should evaluate whether the aforementioned factors could help standardize a *P. vivax* culture.

Other important considerations include knowing whether the abundance of receptors (as has been showed for DARC and CD71) or remodelling other proteins during reticulocyte maturation (as has been shown for DARC [82]) can have an impact on the development of a *P. vivax* in vitro culture.

This review has described different factors affecting *P. vivax* in vitro culture, ranging from using several strains and isolates to different target cell sources and physicochemical variations. Using the same parasite strain and CD71^high^TO^high^ host cells could be a starting point for removing the spoke in the wheel and advance knowledge regarding *P. vivax* biology.

## Abbreviations

AMA-1: apical membrane antigen 1
CD71: cluster of differentiation 71 transferrin receptor
DBP: duffy binding protein
DMEM: Iscove’s Modified Dulbecco’s Medium
EBP-2: erythrocyte binding protein 2
GAMA: GPI-anchored micronemal antigen
RBC: red blood cell
HEPES: (4-(2-hydroxyethyl)-1-piperazineethanesulfonic acid)
HSC: haematopoietic stem cell
MSP-1: merozoite surface protein 1
*Pv*TRAg: *Plasmodium vivax* tryptophan rich antigen
RBP: reticulocyte-binding protein
RBSA: reticulocyte binding surface antigen
RON2: rhoptry neck protein 2
RON4: rhoptry neck protein 4
RON5: rhoptry neck protein 5
RPMI-1640: Roswell Park Memorial Institute medium
UCB: umbilical cord blood
TO: Thiazole Orange
